# Triple Agonism Based Therapies for Obesity

**DOI:** 10.1007/s12170-025-00770-z

**Published:** 2025-07-28

**Authors:** Jonathan Goldney, Malak Hamza, Farhaana Surti, Melanie J. Davies, Dimitris Papamargaritis

**Affiliations:** 1 Diabetes Research Centre, College of Life Sciences, University of Leicester, Leicester General Hospital, Leicester, LE5 4PW UK; 2 NIHR Leicester Biomedical Research Centre, University Hospitals of Leicester NHS Trust and University of Leicester, Leicester, LE5 4PW UK

**Keywords:** Retatrutide, Triple agonist, Glucagon, Obesity, Type 2 diabetes, GLP-1

## Abstract

**Purpose of the Review:**

Glucagon-like peptide 1 (GLP-1) receptor agonists (RA) have transformed obesity and type 2 diabetes (T2D) management. Tirzepatide, the first dual GLP-1/glucose-dependent insulinotropic polypeptide (GIP) RA approved for both conditions, has paved the way for next-generation incretin-based therapies. Among these, triple agonists targeting GLP-1, GIP, and glucagon receptors represent a promising next step. This review outlines the rationale for their development and summarizes clinical trial data, focusing on retatrutide, the most advanced candidate.

**Recent Findings:**

Retatrutide is the first triple agonist (acting on GLP-1/GIP/glucagon receptors) with published phase 2 data in people with obesity as well as in people with T2D. Retatrutide achieved up to 24.2% mean weight loss after 48 weeks in individuals with obesity and 16.9% in those with T2D after 36 weeks. In the T2D study, HbA1c improved by 2.2%, with 82% of participants reaching HbA1c ≤ 6.5%. Retatrutide also improved multiple cardiometabolic parameters, including blood pressure, lipids, waist circumference, and liver fat (82% reduction in hepatic steatosis). Gastrointestinal symptoms were the most common side effects; no major safety concerns were observed. A comprehensive phase 3 program is ongoing to evaluate efficacy, safety, and cardiovascular/renal outcomes in people with obesity and/or T2D. Other unimolecular triple agonists and combination regimens involving tirzepatide with additional mono agonists are also in development.

**Summary:**

Retatrutide, a triple agonist now in phase 3 trials, has the potential to become the most effective pharmacological treatment for obesity while also offering substantial benefits in T2D management and other cardiometabolic risk factors.

## Introduction

Obesity is a chronic progressive disease that increases the risk of multiple obesity-related complications such as cancer and cardiovascular disease and is associated with impaired quality of life, and reduced life expectancy [1]. Despite the increasing prevalence of obesity worldwide, effective long-term treatments remain a challenge [2]. While lifestyle interventions are the cornerstone of obesity prevention and management, they often fail to result in sustained and meaningful weight loss (WL). For decades, bariatric surgery (BS) has been the most effective treatment for severe obesity, resulting in 25–30% WL after the first postoperative year and around 20–25% WL long-term [3]. However, BS remains invasive and is not readily scalable at the population level.

Advances in the understanding of gut hormone physiology– particularly their role in appetite regulation, metabolism, and glucose homeostasis - have driven the development of glucagon-like peptide 1 (GLP-1) receptor agonists (RAs) as effective and safe treatments for type 2 diabetes (T2D) and obesity. GLP-1, a hormone primarily secreted from the ileum in response to nutrient intake, exerts multiple metabolic effects including appetite suppression, delayed gastric emptying, enhanced insulin secretion, and inhibition of glucagon release [4]. Initially approved for T2D management, GLP-1 RAs have also demonstrated cardiovascular and renal benefits [5–7]. In recent years, higher doses of some GLP-1 RAs have been used for obesity treatment, with liraglutide (3 mg) and semaglutide (2.4 mg) showing marked efficacy in inducing WL. In 2021, semaglutide 2.4 mg became the most-effective GLP-1 RA approved for obesity management with 14.9% mean WL at 68 weeks vs. 2.4% with placebo [8]. However, mean WL with GLP-1 RAs remains substantially lower than that achieved with BS and treatment response to GLP-1 RAs is heterogeneous.

Looking for the next step in obesity pharmacotherapies, the concept of combining entero-pancreatic hormones for obesity treatment was supported by the marked WL, sustained weight maintenance, and metabolic benefits observed with BS, which increase multiple gut hormone levels [9–14]. To approach the efficacy of BS, dual and triple agonists combining GLP-1 with other entero-pancreatic hormones with complementary actions, including glucose-dependent insulinotropic polypeptide (GIP), glucagon (GCG) and amylin are currently under development [15]. These therapies aim to enhance WL and improve metabolic outcomes by engaging multiple hormonal pathways, with preclinical and early-phase clinical trials supporting their potential as next-generation obesity treatments [16–23].

Building on the success of tirzepatide—the first approved dual GLP-1/GIP co-agonist—other dual agonists, including those targeting both GLP-1 and GCG receptors, are in an advanced stage of development. In parallel, triple agonists simultaneously targeting GIP, GLP-1, and GCG receptors are also under investigation, aiming to deliver greater WL and metabolic improvements than mono- or dual-agonists.

This review summarizes completed and ongoing clinical trials of triple agonists, with a focus on retatrutide—the most advanced candidate to date. It also discusses key challenges in their development and explores future directions, including clinical considerations and emerging research with triple agonists.

## Stepping-Stones Towards Triple Agonism: A Summary of GLP-1/GIP and GLP-1/GCG Dual-Agonist Development

The development of triple agonists for obesity has built upon successes of several dual combinations of entero-pancreatic hormone receptor agonists, in particular, GLP-1/GIP dual agonists and GLP-1/GCG dual agonists.

### GLP-1/GIP Dual Agonism

**Fig. 1:**
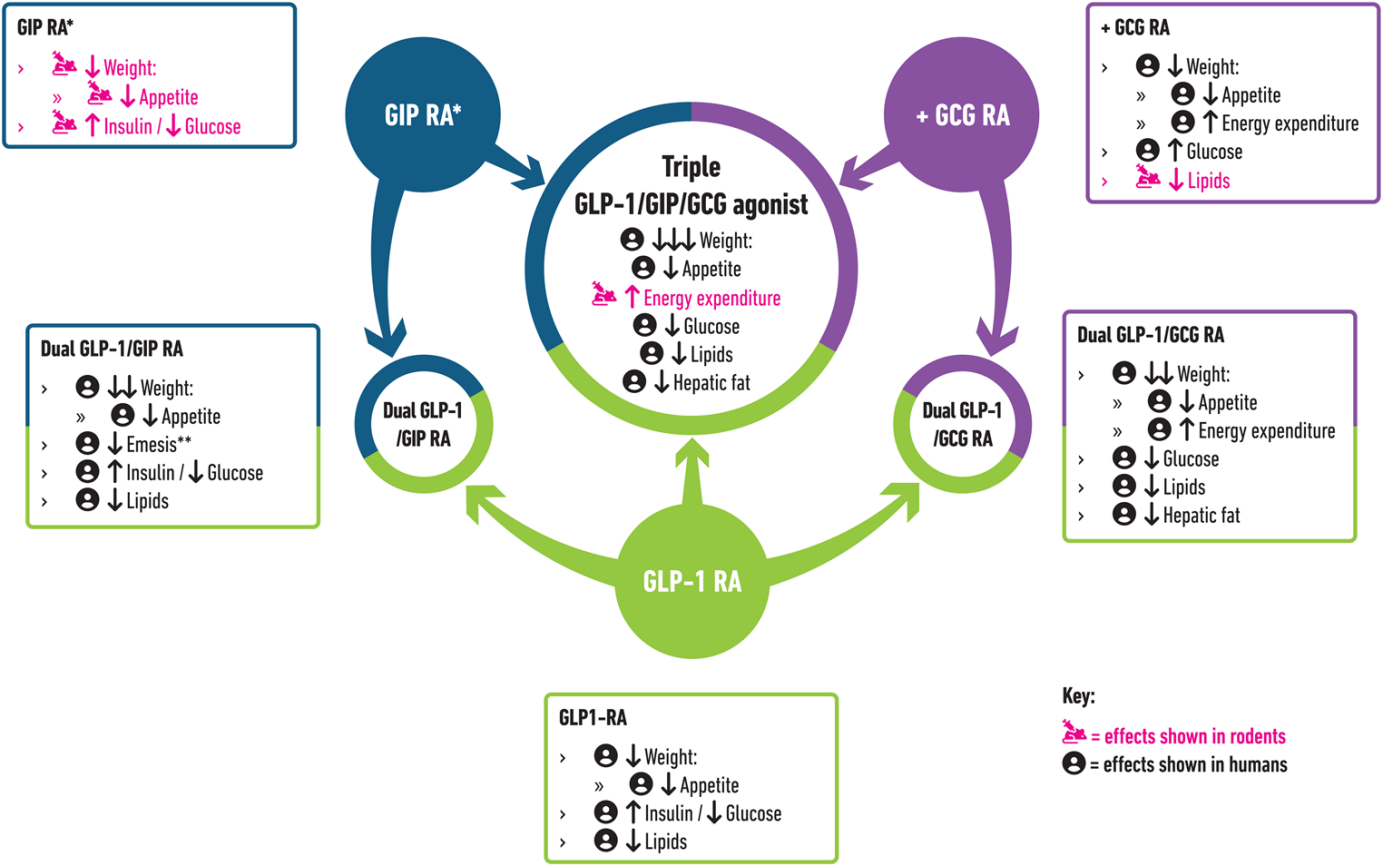
Demonstrated metabolic effects of GIP, GLP-1 and GCG agonism as mono, dual and triple receptor agonists. Arrows represent effects as compared to no treatment/ placebo unless otherwise stated. *Data shown relate to long-acting GIP RA use only. **Decreased emesis as compared to GLP-1RA use alone. GIP: Glucose-dependent insulinotropic polypeptide; GLP-1: Glucagon-like peptide 1; GCG: Glucagon; RA: Receptor agonist

GIP is an entero-pancreatic hormone secreted from K-cells of the duodenum/upper jejunum in response to ingestion of nutrients [24]. GIP stimulates also glucose-dependent insulin release in people without diabetes and may have a role in appetite regulation: despite mixed findings, preclinical research suggests that the addition of GIP RA may enhance appetite suppression and WL associated with a fixed-dose of GLP-1 RA (Fig. 1) [16, 19]. Furthermore, GIP inhibits hindbrain neurons regulating emesis [25] and GIP agonism reduces emesis caused by a GLP-1 RA in both rodent and human studies [25, 26].

Tirzepatide is the only licenced GLP-1/GIP co-agonist for obesity, with several more GLP-1/GIP co-agonists in development [15]. In the SURMOUNT-1 trial, tirzepatide 15 mg weekly achieved 20.9% WL over 72 weeks (vs. 3.1% with placebo) in people with obesity without diabetes, the highest reported among approved obesity pharmacotherapies [27]. Moreover, the SURPASS programme demonstrated that tirzepatide 15 mg is more efficacious in glycaemic control for people with T2D compared to insulin therapies as well as GLP-1 mono-agonists including semaglutide 1 mg and dulaglutide 1.5 mg. Apart from improvements in multiple cardiometabolic risk factors, tirzepatide also improves various obesity-related complications such as obstructive sleep apnoea (OSA), metabolic dysfunction-associated steatohepatitis (MASH) and heart failure with preserved ejection fraction [28–30]. Ongoing studies are evaluating cardiovascular outcomes with tirzepatide in populations with overweight/obesity and T2D (SURPASS-CVOT) and in those with obesity but without diabetes (SURMOUNT-MMO) [28, 29].

### GLP-1/GCG Dual Agonism

GCG is a pancreatic hormone secreted from α-cells. It is historically known for its hyperglycaemic effects and use in treating hypoglycaemia. However, it also reduces appetite and increases energy expenditure [31–33]. Subsequent studies revealed that co-infusion of GLP-1 with GCG can mitigate the GCG-induced hyperglycaemia [34], while preserving GCG’s metabolic benefits, providing a strong rationale for the development of GLP1/GCG co-agonists as obesity treatments (Fig. 1) [15].

Dual GLP-1/GCG agonists such as mazdutide and survodutide are currently in phase 3 clinical trials as obesity treatments, with pemvidutide having recently completed phase 2 trials. A press release from the phase 3 trial data for mazdutide (6 mg weekly for 48 weeks) showed a 14.8% WL (vs. 0.5% WL with placebo) [35], while phase 2 results for survodutide (4.8 mg weekly for 46 weeks) demonstrated an 18.7% reduction in bodyweight (vs. 2.3% with placebo) [36].

Beyond WL, GCG agonism may be particularly beneficial for individuals with metabolic-dysfunction associated steatotic liver disease (MASLD) and MASH. GCG has several established roles in the liver including reducing hepatic lipid accumulation; increasing mitochondrial turnover and function; reducing oxidative stress; and reducing stellate cell activation, potentially reducing their fibrotic response to liver injury [37]. Indeed, GCG antagonists, developed as potential glucose-lowering therapies, were associated with an increase in hepatic steatosis [38], suggesting that GCG agonists may reduce hepatic steatosis [39]. Supporting this, in a phase 2a trial in people with MASLD, the dual GLP-1/GCG agonist efinopegdutide (10 mg) reduced liver fat by 72.7%, compared to 42.3% with semaglutide (1 mg) despite similar WL—indicating potential weight-independent effects of GCG on hepatic steatosis [40]. Additionally, in phase 2 studies in people with MASH and liver fibrosis, survodutide not only significantly improved liver steatosis, but also led to histologic improvement of MASH without worsening fibrosis in 43–62% of participants, versus 14% with placebo [41].

## GLP-1/GIP/GCG Triple Agonists

The most researched combination of receptors targeted with triple agonists are GLP-1/GIP/GCG. The success of GLP-1/GIP and GLP-1/GCG dual agonists thus far suggests that GLP-1/GIP/GCG may provide even greater WL compared to dual agonist therapies. Indeed, in rodent studies, GLP-1/GIP/GCG triple agonists produced greater WL than mono- or GLP-1/GIP dual agonists [22], suggesting potential additional metabolic benefits **(**Fig. 1**)**.

## Retatrutide

### Background

Retatrutide (LY3437943) is the first GLP-1/GIP/GCG triple agonist to complete phase 2 trials, with phase 3 studies ongoing for obesity and/or T2D management. It is a 39 amino-acid, single peptide, engineered from a GIP peptide backbone, conjugated to a C20 fatty di-acid moiety to enable albumin binding and extend its half-life [42]. Retatrutide has ≈ 2.5-times lower potency at the GLP-1 receptor than human GLP-1, 8.9-times higher potency at the GIP-receptor than human GIP and 2.9-times lower potency at the GCG-receptor than human GCG [42].

### Preclinical Studies

In preclinical trials, retatrutide was administered to male mice models with obesity [42]. The results showed that retatrutide significantly delayed gastric emptying, reduced food intake, and caused dose-dependent WL. The WL was primarily due to reductions in fat mass with less impact on lean mass. Additionally, retatrutide lowered blood glucose and insulin levels, suggesting that retatrutide enhanced insulin sensitivity. Retatrutide further reduced plasma alanine aminotransferase and liver triglycerides, suggesting improvements in liver health [42]. Further studies demonstrated that retatrutide also increased energy expenditure through GCG receptor activation [42]. These findings highlighted retatrutide’s potential as a potent therapeutic agent for obesity and metabolic disorders.

### Phase 1 Trials

In a first-in-human, randomized, placebo-controlled, single ascending dose, phase 1 trial in healthy participants, retatrutide demonstrated a favourable safety profile (similar to other incretin-based therapies) and early signs of efficacy with dose-dependent WL [42]. The pharmacokinetic profile supported once-weekly dosing, with a mean half-life of approximately six days. Appetite suppression was observed at doses ≥ 0.3 mg while significant reductions in circulating triglycerides, branched-chained amino-acids and fasting and postprandial glucagon levels were also seen, along with increasing insulin secretion.

Building on these results, a phase 1b trial in individuals with T2D further evaluated the safety, pharmacokinetics, and pharmacodynamics of once-weekly retatrutide over 12 weeks [43]. The medication produced dose-dependent improvements in both glycaemic control and body weight. At the highest dose (3/6/9/12 mg escalation scheme), mean HbA1c was reduced by up to 1.6% and weight by nearly 9 kg, which was significantly greater than placebo. Retatrutide also lowered fasting and postprandial glucose, reduced appetite, and improved lipid profiles, including reductions in low-density lipoprotein (LDL) cholesterol and triglycerides. Adverse events were mostly mild-to-moderate gastrointestinal symptoms, consistent with other incretin-based therapies. A substudy of this phase 1b trial showed also that retatrutide delays gastric emptying in humans, as measured by the acetaminophen test, though this effect lessens over time due to tachyphylaxis [44].

### Phase 2 Trials

The findings of phase 1 studies supported the continued clinical development of retatrutide as a promising candidate for the treatment of obesity and T2D. Two phase II trials - one in a population with obesity (without T2D) [45], and a second in a population with T2D and overweight/obesity [46], have recently been published and their key findings are presented below (Table 1).

**Table 1:** Key efficacy outcomes from the phase 2 studies of retatrutide in individuals with obesity and with type 2 diabetes

Outcome	Obesity trial (48 weeks)							Type 2 diabetes trial (36 weeks)							
	Placebo *N* = 70	Retatrutide dose (initial dose)						Placebo *N* = 45	Retatrutide dose (initial dose)						Dulaglutide 1.5 mg *N* = 46
		1 mg (1mg) *N* = 69	4 mg (2mg) *N* = 33	4 mg (4mg) *N* = 34	8 mg (2mg) *N* = 35	8 mg (4mg) *N* = 35	12 mg (2mg) *N* = 62		0.5 mg (0.5mg) *N* = 47	4 mg (2mg) *N* = 23	4 mg (4mg) *N* = 24	8 mg (2mg) *N* = 26	8 mg (4mg) *N* = 24	12 mg (2mg) *N* = 46	
**Anthropometric outcomes**															
Change in body weight (%)	−2.1 (− 3.5, − 0.7)	−8.7 (− 10.5, − 6.8)	−16.3 (− 19.4, − 13.2)	−17.8 (− 20.8, − 14.8)	−21.7 (− 24.5, − 19.0)	−23.9 (− 26.8, − 20.9)	−24.2 (− 26.6, − 21.8)	-3.0 (0.9)	-3.2 (0.6)	-7.9 (1.3)	-10.4 (1.6)	-16.8 (1.6)	-16.3 (1.7)	-16.9 (1.3)	-2.0 (0.7)
Difference in change in body weight from placebo (%)	—	−6.6 (− 8.9, − 4.2)	−14.2 (− 17.6, − 10.8)	−15.7 (− 19.1, − 12.4)	−19.6 (− 22.7, − 16.5)	−21.8 (− 25.1, − 18.5)	−22.1 (− 24.9, − 19.3)	—	-0.2 (-2.2, 1.9)	-4.9 (-8.0, -1.8)	-7.4 (-10.8, -3.9)	-13.8 (-17.4, -10.3)	-13.3 (-17.0, -9.7)	-13.9 (-17.0, -10.8)	—
Weight reduction ≥ 15%	2	16	55	64	73	77	83	2	0	10	26	57	63	58	0
Weight reduction ≥ 20%	1	6	31	29	50	70	64	2	0	9	14	39	39	40	0
Weight reduction ≥ 25%	0	6	13	19	36	43	48	NR	NR	NR	NR	NR	NR	NR	NR
Weight reduction ≥ 30%	0	1	6	10	16	17	26	NR	NR	NR	NR	NR	NR	NR	NR
Change in WC (cm)	−2.6 (− 4.6, − 0.7)	−6.5 (− 8.7, − 4.3)	−14.6 (− 17.6, − 11.5)	−14.9 (− 18.2, − 11.5)	−18.5 (− 21.4, − 15.7)	−18.5 (− 21.5, − 15.5)	−19.6 (− 22.1, − 17.1)	-0.9 (1.1)	-2.2 (1.0)	-4.6 (1.8)	-4.2 (1.9)	-12.0 (1.9)	-12.0 (1.4)	-13.2 (2.8)	-2.2 (0.8)
**Other metabolic outcomes**															
Change in HbA1c (%)	0.0 (-0.1, 0.1)	-0.2 (-0.2, -0.1)	-0.2 (-0.3, -0.1)	-0.3 (-0.4, -0.3)	-0.5 (-0.6, -0.3)	-0.5 (-0.5, -0.4)	-0.4 (-0.5, -0.4)	−0.3 (0.2)	−0.5 (0.2)	−1.3 (0.2)	−1.5 (0.2)	−2.1 (0.2)	−1.9 (0.2)	−2.2 (0.1)	−1.4 (0.1)
Change in SBP (mmHg)*	-2.9 (-5.4, -0.4)	-4.8 (-7.2, -2.3)	-8.7 (-12.7, -4.8)	-8.3 (-11.5, -5.1)	-8.8 (-11.6, -6.0)	-11.8 (-14.8, -8.8)	-8.8 (-11.9, -5.8)	1.5 (2.1)	−2.8 (1.5)	−4.0 (2.5)	−6.5 (2.5)	−5.9 (3.0)	−8.3 (2.5)	−8.8 (1.5)	−1.5 (1.9)
Change in DBP (mmHg)*	-1.0 (-2.6, 0.5)	-2.2 (-4.0, -0.5)	-3.2 (-6.0, -0.4)	-2.9 (-5.0, -0.8)	-3.4 (-5.1, -1.7)	-3.5 (-5.6, -1.4)	-2.8 (-4.6, -0.9)	−1.2 (1.0)	−1.6 (1.0)	−2.9 (1.1)	−2.0 (1.3)	−3.4 (1.4)	−3.5 (1.4)	−3.9 (0.9)	0.0 (1.2)
Change in triglycerides (%)	1.4 (-9.3, 12.1)	-17.9 (-25.1, -10.8)	-33.0 (-39.4, -26.5)	-34.9 (-46.2, -23.6)	-43.6 (-50.1, -37.1)	-37.2 (-44.5, -29.9)	-39.9 (-46.7, -33.1)	−9.9 (5.3)	−14.0 (5.3)	−11.6 (7.3)	−9.8 (7.2)	−35.0 (7.5)	−32.0 (6.7)	−34.4 (7.6)	−4.3 (7.9)
Change in total cholesterol (%)	1.9 (-1.5, 5.2)	-4.5 (-8.0, -1.0)	-12.6 (-17.0, -8.3)	-10.0 (-15.8, -4.3)	-18.2 (-22.2, -14.1)	-13.9 (-17.8, -9.9)	-17.8 (-21.5, -14.2)	−2.2 (5.5)	−7.8 (5.5)	−7.0 (7.3)	−7.1 (6.5)	−16.7 (7.1)	−11.9 (7.1)	−14.8 (7.5)	−0.9 (6.9)
Change in LDL cholesterol (%)	-0.3 (-5.0, 4.4)	-4.7 (-9.3, -0.1)	-14.5 (-20.7, -8.3)	-10.2 (-17.6, -2.8)	-20.7 (-26.1, -15.3)	-16.8 (-22.2, -11.5)	-21.7 (-27.2, -16.2)	−2.8 (5.4)	−10.2 (5.5)	−6.2 (6.8)	−7.4 (6.5)	−12.5 (6.9)	−11.9 (6.5)	−6.9 (6.5)	0.5 (6.6)
Change in heart rate (beats/min)	0.9 (-1.0, 2.9)	2.7 (0.8, 4.7)	3.6 (0.8, 6.4)	2.6 (0.4, 4.8)	5.6 (3.1, 8.1)	3.9 (1.5, 6.4)	6.7 (4.6, 8.8)	−3.2 (1.0)	1.5 (1.1)	0.0 (1.6)	2.0 (1.1)	1.3 (1.8)	4.3 (1.7)	3.9 (1.4)	1.8 (1.3)

Values presented as Least-squares mean (95% confidence interval [two numbers] or standard error [single number]) for continuous variables and as percentage of total for categorical variables

Results represent changes from baseline to 48 weeks in the obesity trial, *excluding blood pressure which was change to 36 weeks; and change from baseline to 36 weeks in the type 2 diabetes trial. All findings were reported as efficacy end points, using the data from all the participants who underwent randomization, excluding those who discontinued treatment because of inadvertent enrolment.

**WC**: Waist circumference; **SBP**: Systolic blood pressure; **DBP**: Diastolic blood pressure; **LDL**: Low density lipoprotein; **NR**: Not reported

Data from: [45, 46]

#### Design

In the obesity trial, 338 participants (mean age 48 years, 48% women, mean BMI 37.3 kg/m^2^) were randomised (2:1:1:1:1:2:2) to retatrutide 1 mg, 4 mg (titration), 4 mg (no titration), 8 mg (slow titration), 8 mg (fast titration), 12 mg or placebo over 48 weeks [45]. All participants received a lifestyle intervention involving regular counselling regarding diet and physical activity. The primary endpoint was change in bodyweight from baseline at 24 weeks, with 48 weeks included as a secondary outcome.

The trial in people with T2D included 281 participants (mean age 56 years, 56% women, mean BMI 35.0 kg/m^2^, mean HbA1c 8.3%) treated with lifestyle only and/or metformin, with similar intervention arms to the obesity trial, albeit retatrutide 0.5 mg rather than 1 mg, and dulaglutide 1.5 mg was an additional comparator [46]. The trial duration was 36 weeks. The primary endpoint was HbA1c change from baseline to 24 weeks, and secondary endpoints included change in HbA1c and bodyweight at 36 weeks.

#### Weight Loss

In both trials, a marked dose-dependent WL was observed. In the obesity trial, maximal WL was achieved with retatrutide 12 mg after 48 weeks: -24.2% vs. -2.1% with placebo, with 64% vs. 1% achieving ≥ 20% WL respectively (Table 1) [45]. Greater WL was seen in women with higher doses of retatrutide (-28.5% WL vs. -21.9% WL in men) and in those with baseline BMI ≥ 35 kg/m^2^ (-26.5% WL vs. -22.1% WL with BMI < 35 kg/m^2^).

In the T2D trial, WL was lower than in the obesity trial however the trial duration was shorter (36 weeks) [46]. Participants receiving retatrutide 12 mg experienced − 16.9% WL vs. -3.0% with placebo and − 2.0% with dulaglutide 1.5 mg. These findings highlight the substantial weight-lowering effect of retatrutide, even in a relatively short treatment period and in people with T2D.

#### Other Metabolic Benefits

In the T2D trial, there were also dose-response reductions in HbA1c: retatrutide 12 mg reduced HbA1c by 2.16% at 36 weeks, corresponding to an estimated difference from dulaglutide 1.5 mg of -0.80% (95% CI: -1.16, -0.44); and estimated difference from placebo of -1.85% (-2.39, -1.31) [46]. Moreover, a greater proportion of participants on retatrutide 8 and 12 mg (77–82%) achieved an HbA1c ≤ 6.5% compared to placebo (5%) and dulaglutide 1.5 mg (43%). The average HbA1c reduction may have been attenuated by ‘floor effect’, as a high proportion of participants approached near-normal glycaemic levels.

Both trials also showed dose-dependent and marked improvements in waist circumference, lipids and blood pressure (Table 1). Furthermore, a substudy of the obesity trial investigated the mean relative change from baseline in liver fat at 24 and 48 weeks in 98 participants with MASLD at baseline. There was a dose-response reduction in liver fat from baseline at 24 weeks with increasing retatrutide doses: -82.4% with 12 mg retatrutide vs. + 0.3% with placebo [47]. At 24 weeks, healthy liver fat levels (< 5%) were achieved by 86% of participants on retatrutide 12 mg and 0% in the placebo group.

A post-hoc analysis of the phase 2 trials also evaluated retatrutide’s impact on kidney function in individuals with T2D or obesity, most of whom had normal kidney function (6–33% had albuminuria; 0–9% had eGFR < 60 mL/min/1.73 m² across trials and arms) [48]. In participants with T2D, retatrutide 12 mg reduced urine albumin-creatinine ratio (UACR) by 37%, however eGFR was unchanged. In those with obesity, retatrutide 12 mg reduced UACR by 31.5% and increased eGFR by 8.5 mL/min/1.73 m². While the clinical relevance of these findings in a population largely free from kidney disease remains uncertain, the reductions in albuminuria, blood pressure, and the favourable safety profile support further investigation of retatrutide’s potential kidney-protective effects in high-risk populations. Indeed, a phase 2 mechanistic trial (NCT05936151) investigating change in eGFR from baseline to week 24 with multiple doses of retatrutide vs. placebo in 120 people with overweight/obesity and chronic kidney disease (CKD) is ongoing.

#### Adverse Events

In both phase 2 trials, there were no major safety signals with retatrutide use [45, 46]. Side effects were mainly mild-to moderate gastro-intestinal symptoms (nausea, vomiting, diarrhoea) mostly occurring with higher doses of retatrutide and with faster titration (Table 2).

**Table 2:** Key safety outcomes from the phase 2 studies of retatrutide in individuals with obesity and with type 2 diabetes

Outcome	Obesity trial (48 weeks)							Type 2 diabetes trial (36 weeks)							
	Placebo *N* = 70	Retatrutide dose (initial dose)						Placebo *N* = 45	Retatrutide dose (initial dose)						Dulaglutide 1.5 mg *N* = 46
		1 mg (1mg) *N* = 69	4 mg (2mg) *N* = 33	4 mg (4mg) *N* = 34	8 mg (2mg) *N* = 35	8 mg (4mg) *N* = 35	12 mg (2mg) *N* = 62		0.5 mg (0.5mg) *N* = 47	4 mg (2mg) *N* = 23	4 mg (4mg) *N* = 24	8 mg (2mg) *N* = 26	8 mg (4mg) *N* = 24	12 mg (2mg) *N* = 46	
Any adverse event during treatment	70	84	73	85	80	94	92	62	55	57	79	73	71	76	67
Serious adverse event	4	4	0	6	3	6	3	7	6	4	8	8	4	4	2
Adverse events leading to discontinuation	0	7	6	9	14	6	16	4	2	0	4	12	17	15	2
**Selected adverse events**															
Nausea	11	14	18	36	17	60	45	4	4	9	25	27	42	20	17
Decreased appetite	9	13	18	24	11	31	29	0	4	4	21	19	17	20	13
Diarrhoea	11	9	12	12	20	20	15	4	2	9	25	19	29	15	9
Vomiting	1	3	12	12	6	26	19	2	2	4	0	8	17	11	9
Constipation	3	7	15	6	11	11	16	2	6	9	17	12	8	11	7
Cardiac arrhythmia*	3	4	0	6	0	14	11	2	4	4	4	8	8	7	4
Hepatic or biliary disorders**	3/0	7/0	3/0	0/0	3/3	6/6	3/0	2	0	0	4	4	4	0	0
Hypoglycaemia (< 54 mg/dL or severe)	NA	NA	NA	NA	NA	NA	NA	0	0	0	4	4	0	2	0
Pancreatitis	0	0	0	0	0	0	2	0	2	0	0	4	0	0	0

Values presented as percentage of total

Adverse events are presented for all participants who were randomised and took at least one dose of treatment, regardless of whether they discontinued

* For the T2D trial, this was limited to ‘supraventricular arrhythmias and cardiac conduction disorders’

** For the obesity trial these are presented as hepatic disorder / biliary disorder separately

**NA**: Not applicable

Data from: [45, 46]

In the obesity trial, serious adverse events (SAEs) occurred in 4% of participants with retatrutide use overall (0–6% across groups) vs. 4% with placebo. Moreover, adverse events leading to medication discontinuation were reported by 10% in the overall retatrutide groups (6–16% across groups vs. 0% with placebo).

The T2D trial showed a similar safety profile, with SAEs occurring in 4–8% of participants at the retatrutide groups vs. 7% with placebo and 2% with dulaglutide. Rates of treatment discontinuation varied across dosing regimens from 0 to 17% (vs. 4% with placebo and 2% with dulaglutide).

#### Ongoing Phase 3 Trials

**Fig. 2:**
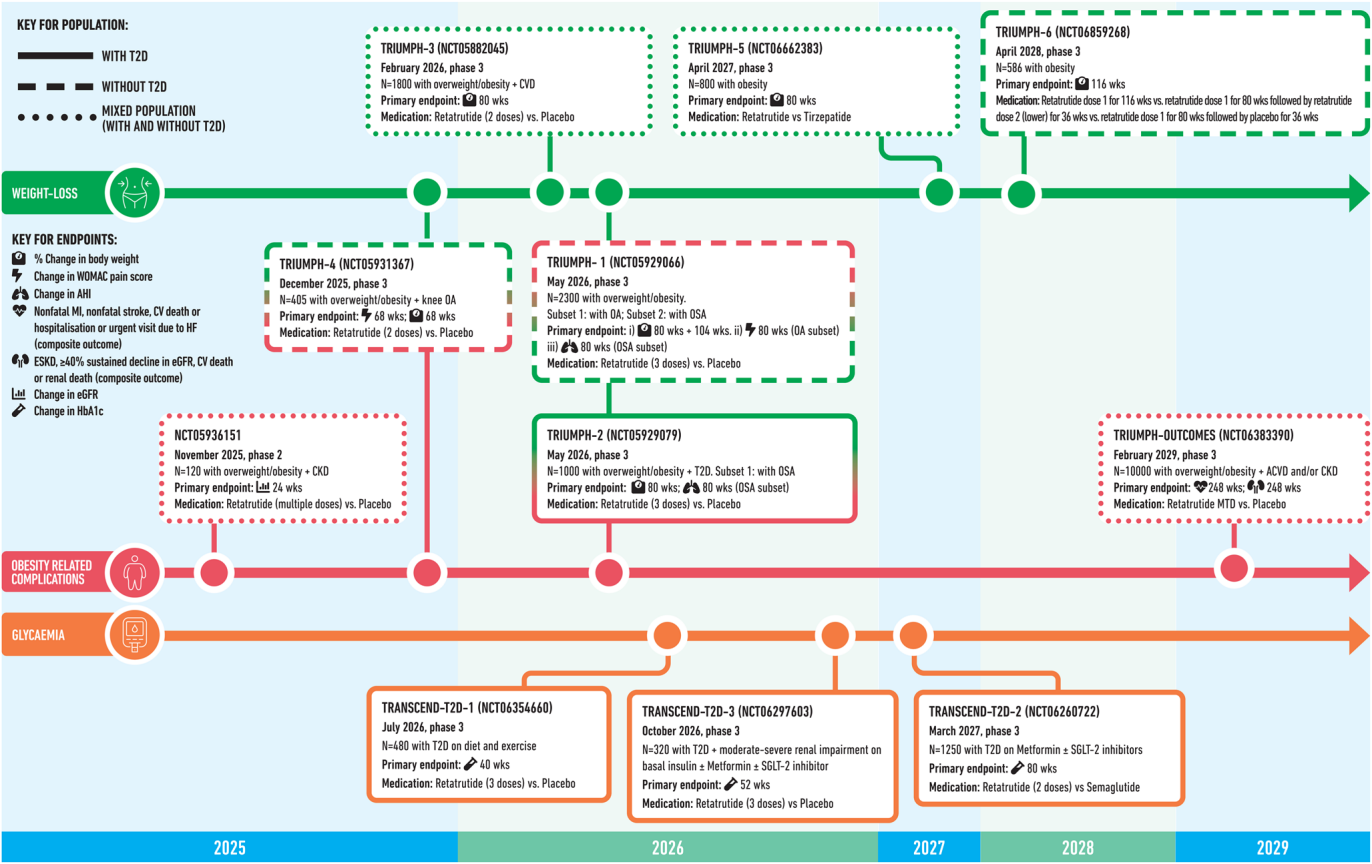
The pipeline of phase 2 and phase 3 trials investigating the efficacy and safety of retatrutide. **T2D**: Type 2 diabetes; **SGLT-2**: Sodium-glucose co-transporter-2; **OA**: Osteoarthritis; **OSA**: Obstructive sleep apnoea; **WOMAC**: Western Ontario and McMaster Universities Osteoarthritis Index; **AHI**: Apnoea-Hypopnea Index; **MTD**: Maximum tolerated dose; **ESKD**: End-stage kidney disease; **eGFR**: estimated glomerular filtration rate; **CV**: Cardiovascular; **CVD**: Cardiovascular disease; **ASCVD**: Atherosclerotic cardiovascular disease; **BMI**: Body mass index; **MI**: Myocardial infarction; **CKD**: Chronic kidney disease.

Several phase 3 trials are underway investigating the efficacy of retatrutide in larger populations (Fig. 2). The TRIUMPH programme will investigate retatrutide in people with obesity and the TRANSCEND-T2D programme is investigating retatrutide in people with T2D and overweight/obesity.

TRIUMPH-1 (NCT05929066) will look at the efficacy and safety of retatrutide in people with overweight/obesity without T2D and will include subsets of participants with knee osteoarthritis (OA) or OSA. The primary endpoint will be bodyweight change (week 80 and 104), change in Western Ontario and McMaster Universities Osteoarthritis Index (WOMAC) in the OA subset, and change in Apnoea-Hypopnea Index (AHI) in the OSA subset.

TRIUMPH-2 (NCT05929079) will assess bodyweight change in people with overweight/obesity and T2D and include a subset of participants with OSA, in which AHI will also be investigated as a primary endpoint. TRIUMPH-3 (NCT05882045) will investigate change in bodyweight in people with overweight/obesity and cardiovascular disease, and TRIUMPH-4 (NCT05931367) will assess change in WOMAC score and bodyweight in individuals with overweight/obesity and knee OA.

TRIUMPH-5 (NCT06662383) will compare the efficacy of retatrutide to tirzepatide on bodyweight over 80 weeks in individuals with obesity. Given that obesity is a chronic and relapsing disease, it is important to understand the effectiveness of different strategies to maintain WL: TRIUMPH-6 (NCT06859268) will assess changes in bodyweight at 116 weeks in individuals with obesity who have completed 80 weeks of retatrutide, comparing continued treatment at high or low doses of retatrutide versus switching to placebo for the last 36 weeks. TRIUMPH-OUTCOMES (NCT06383390) will investigate the impact of retatrutide vs. placebo on the time to occurrence of a composite cardiovascular outcome (nonfatal myocardial infarction, nonfatal stroke, cardiovascular death, hospitalization or urgent visit due to heart failure) and a composite renal outcome (end-stage kidney disease, ≥ 40% sustained decline in eGFR, cardiovascular death or renal death) in 10,000 individuals with atherosclerotic cardiovascular disease and/or CKD.

A series of trials in populations with T2D will also be undertaken. TRANSCEND-T2D-1 (NCT06354660) will compare the efficacy and safety of retatrutide compared to placebo in people with inadequately controlled T2D treated with diet and exercise over a period of 11 months. The primary endpoint will be HbA1c change. Investigating the same primary endpoint, TRANSCEND-T2D-2 (NCT06260722) will compare retatrutide to semaglutide in people with inadequately controlled T2D (treated with oral glucose-lowering treatments) over 26 months. Finally, TRANSCEND-T2D-3 (NCT06297603) will compare retatrutide to placebo over 14 months, in people with inadequately controlled T2D and moderate-or-severe renal impairment on basal insulin.

#### The Potential of Retatrutide

Retatrutide leads to 24.2% mean WL at the highest dose after 48 weeks in people with obesity, with no evidence of plateau and up to 26% of participants achieving ≥ 30% WL - approaching the efficacy Roux-en-y gastric bypass, one of the most effective BS procedures. In the T2D phase 2 trial ≈ 17% WL was observed after just 36 weeks of treatment with the highest dose, also without evidence of plateau.

**Fig. 3:**
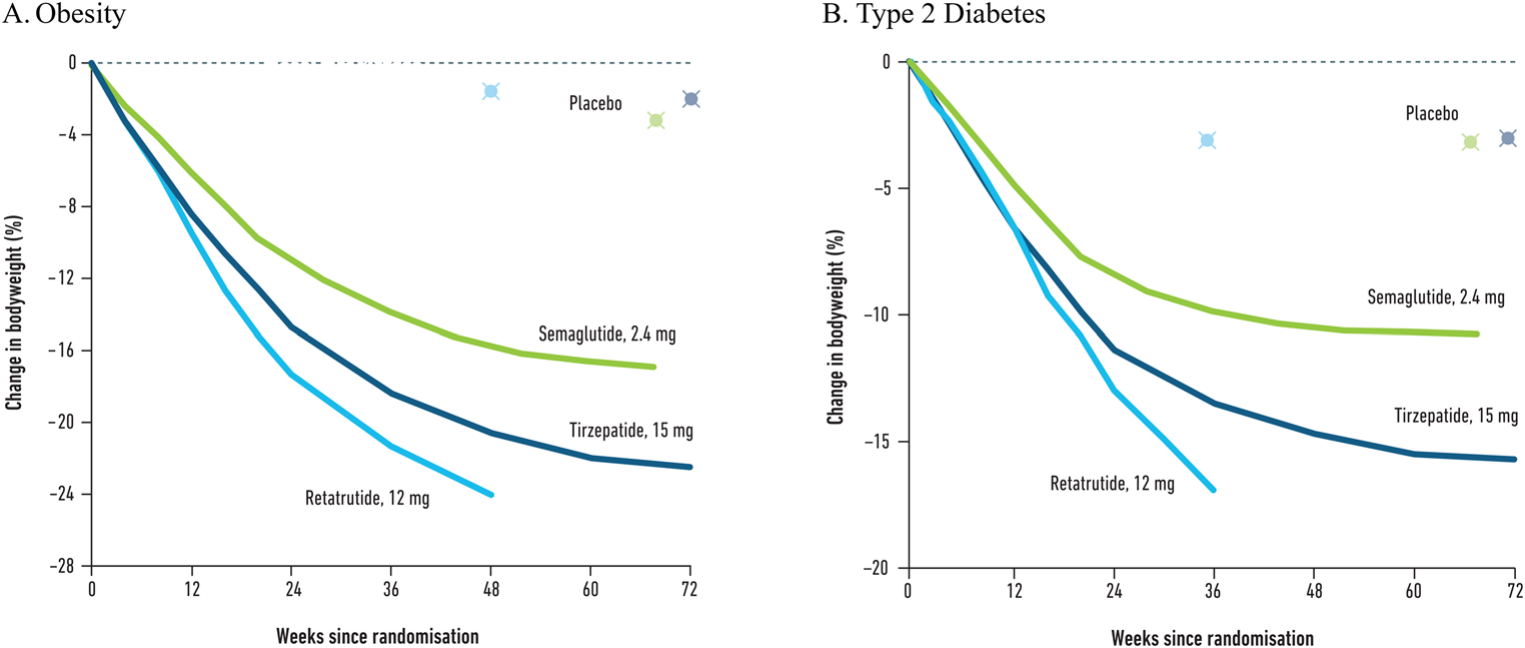
Weight loss with retatrutide as compared to currently licenced obesity pharmacotherapy. Data from: [8, 27, 45, 49, 46, 50]

**Fig. 4:**
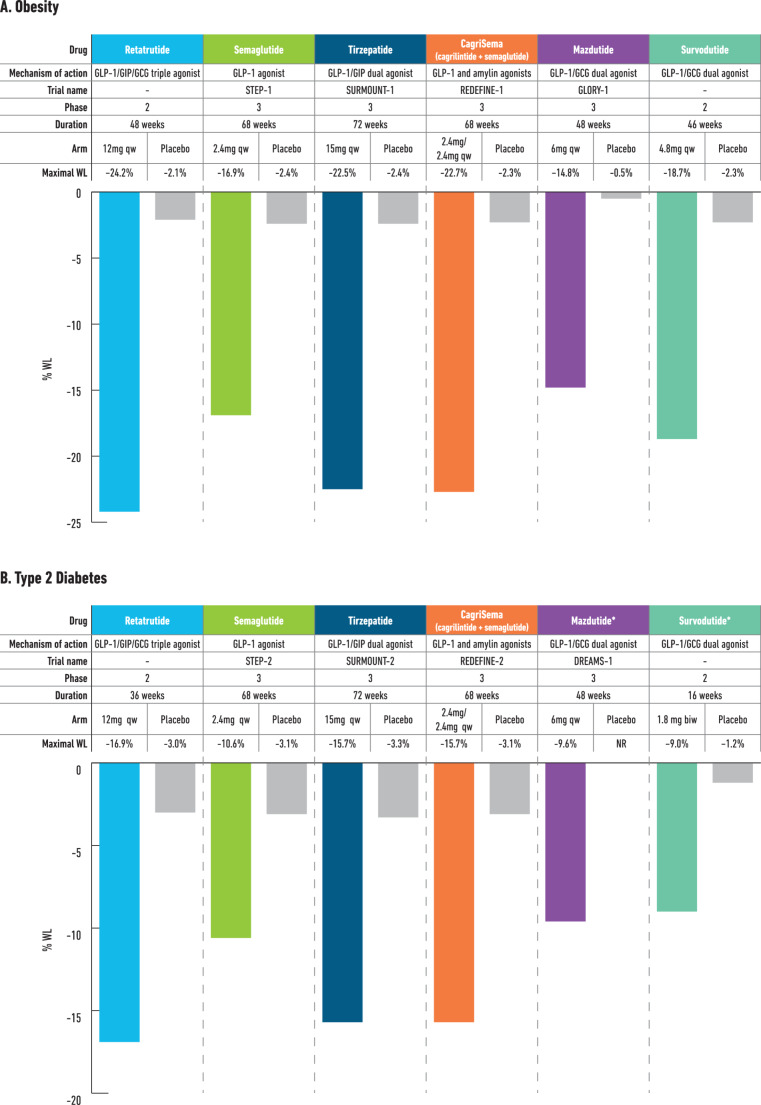
Weight loss with retatrutide as compared to licenced obesity pharmacotherapies and upcoming dual agonists in phase 3 trials in people with obesity and with type 2 diabetes. **GIP**: Glucose-dependent insulinotropic polypeptide; **GLP-1**: Glucagon-like peptide 1; **GCG**: Glucagon; **RA**: Receptor agonist; **qw**: once weekly dosing; **biw**: twice weekly dosing. Data from: [45, 50, 27, 8, 46, 49, 51, 35, 52, 36, 53]

Although no data from head-to-head trials comparing retatrutide to other currently approved obesity treatments exist, data from the phase 2 studies with retatrutide suggest that at higher doses, retatrutide could lead to greater WL compared to the most efficacious approved treatments for obesity and T2D such as tirzepatide 15 mg and semaglutide 2.4 mg (Fig. 3) [8, 27, 45, 46, 49, 50]. Notably, nearly 50% of the retatrutide phase 2 obesity trial were men, which is higher than in phase 3 clinical trials with tirzepatide (32.5% in SURMOUNT-1 [27]) and semaglutide 2.4 mg (25.9% in STEP-1 [8]). Given these differences in cohort characteristics, and that women appeared to experience greater WL than men with retatrutide, it may suggest that the mean WL might have been even higher with a higher proportion of women in phase 2 retatrutide trials. Moreover, the magnitude of WL with retatrutide also exceeded that seen with other pipeline multi-agonists such as CagriSema (cagrilintide, an amylin agonist, in combination with semaglutide), mazdutide and survodutide (Fig. 4) [8, 27, 35, 36, 45, 46, 49–53]. The TRIUMPH-5 study will provide data for a direct comparison between tirzepatide and retatrutide for WL.

Retatrutide also shows great potential for treating T2D– in the phase 2 trial, it showed superior efficacy to dulaglutide 1.5 mg, a widely used GLP-1 RA with proven cardiovascular benefits, in reducing HbA1c and bodyweight. After just 36 weeks, 77–82% of participants on retatrutide achieved euglycaemia (HbA1c ≤ 6.5%) and 57–63% achieved ≥ 15% WL. Notably, the SOS study showed that in people with T2D, achieving and maintaining ≥ 15% WL over 15 years was associated with reductions in both micro- and macrovascular complications following BS [54]. Reassuringly, only a very small percentage of people with T2D experienced hypoglycaemia in the phase 2 trial which included people on lifestyle interventions or metformin; however, the TRANSCEND-T2D-3 study which includes people with T2D and CKD on basal insulin, will provide further insight into hypoglycaemia risk in high-risk populations.

More broadly, WL through any means has the potential to reduce the severity of obesity-associated complications [55]. Indeed, phase 3 trials are evaluating retatrutide in populations with T2D, OSA, knee OA, CKD and cardiovascular disease who may particularly benefit. Similarly, the substudy of the obesity phase 2 trial showed clear promise for retatrutide in the treatment of MASLD [47], and through its GCG receptor agonism, retatrutide may offer benefits beyond WL, through weight-independent metabolic effects at the liver [40]. These benefits may extend to individuals with MASH, however there are currently no retatrutide trials planned in populations with MASLD/MASH.

Additionally, retatrutide markedly improved lipid profiles in people with T2D or obesity, reducing total cholesterol (~ 15–18%), LDL (~ 12–22%), and triglycerides (~ 35–40%)—surpassing dulaglutide 1.5 mg. Beyond WL, these effects may be partly driven by its triple agonism, particularly glucagon receptor activation, which enhances lipid oxidation and reduces hepatic lipid synthesis [56].

### Safety Considerations and Future Outlook

As with all pharmacotherapy for obesity and T2D, establishing cardiovascular safety and demonstrating improvements in meaningful clinical outcomes will be essential, particularly as cardiovascular outcome trials have not yet been completed with multi-agonists (although data are expected soon for tirzepatide: SURPASS-CVOT [T2D] in 2025; SURMOUNT-MMO [without T2D] in 2027). Until such evidence is available for retatrutide, therapies with established cardiovascular and renal benefits—such as semaglutide, supported by SUSTAIN-6 [5] and FLOW [57] trials in people with T2D, and the SELECT trial [58] in people with obesity - should be prioritised in populations with existing cardio-renal disease. The ongoing TRIUMPH OUTCOMES trial will be key in determining the long-term cardio-renal effects of retatrutide in these populations.

Another consideration for GLP-1/GIP/GCG agonists is their potential negative impact on lean mass [32]. GCG can suppress circulating amino-acids [59] and increase protein catabolism, mechanisms thought to contribute to elevated energy expenditure and WL [32, 33, 60]. An extreme example of the effects of GCG on lean mass is in individuals with glucagonoma, which is characterised by very high levels of circulating GCG, low levels of amino-acids, and sarcopenia [61, 62]. Preclinical evidence suggests that increasing protein intake may offset circulating amino-acids reduction, however this may also blunt the increase in whole-body energy expenditure [63]. In the phase 2 trial of retatrutide in individuals with T2D and overweight/obesity, retatrutide caused large reductions in circulating amino-acids, although lean mass and physical function were not directly assessed [46]. However, reassuringly, in the obesity phase 2 trial, self-reported physical function improved with all doses of retatrutide [45]. Additionally, the phase 2 MOMENTUM clinical trial of pemvidutide (a dual GLP-1/GCG agonist) showed that only 21.9% of WL was attributable to reductions in lean mass at 48 weeks (overall WL 10.3–15.6% across doses) [64]. Nonetheless, further data are needed to assess the safety of retatrutide on muscle mass, particularly in populations at risk for sarcopenia, where caution is warranted.

#### Other GLP-1/GIP/GCG Triple Agonists in Development

Besides retatrutide, several GLP-1/GIP/GCG triple agonists are in early-stage clinical trials (Table 3), though the WL efficacy associated with these medications remains unknown. All medications within these trials are once weekly subcutaneously-administered injections. These agents are likely to vary widely in their affinity and potency at each hormone receptor.

**Table 3:** Planned or ongoing phase 2 trials involving GLP1/GIP/GCG triple agonists for obesity and/or type 2 diabetes

Trial	Medications	Population	Number of participants (estimated)	Primary endpoint	Expected completion date	Trial duration	Sponsor
NCT05936151	Retatrutide vs. Placebo	Overweight/Obesity + CKD +/- T2D	120	Change in baseline eGFR	2025-11	18 months	Eli Lilly and Company
CTR20250029	UBT251 4 escalating dose regimes ranging from 0.5-6 mg once weekly vs. Placebo	T2D treated with lifestyle intervention ± metformin	NR	NR	No current end date, Not yet recruiting	24 weeks	Federal Biotechnology/United Bio-Technology
CTR20250212	MWN-101 3 cohorts (4.8 mg, 9.6 mg, 19.2 mg) vs. Placebo	Overweight/Obesity + OSA	108	NR	No current end date, Not yet recruiting	32 weeks	Shanghai Minwei Biotechnology
CTR20240817	MWN-101 4 cohorts, escalating dose to 4.8-19.2 mg vs. Placebo and vs. Semaglutide (0.25-1 mg)	T2D treated with diet and exercise	107	NR	2025-01	12 weeks	Shanghai Minwei Biotechnology
CTR20240802	MWN-101 5 cohorts, escalating dose to 4.8-24 mg target vs. Placebo	Overweight/Obesity ± prediabetes, OSA, obesity related joint pain or dyspnoea	108	NR	2024-11	24 weeks	Shanghai Minwei Biotechnology
NCT04505436	Efocipegtrutide (HM-15211) vs. Placebo	Non-cirrhotic NASH	240	Overall histological resolution of steatohepatitis on overall without worsening of liver fibrosis	2026-11	12 months	Hanmi Pharmaceutical Company Limited

Data sourced from clinicaltrials.gov; www.chinadrugtrials.org.cn

**T2D**: Type 2 diabetes; **OSA**: Obstructive sleep apnoea; **eGFR**: estimated glomerular filtration rate; **CKD**: Chronic kidney disease; **NASH**: Non-alcoholic (metabolic dysfunction) associated steatohepatitis; **NR**: Not reported

However, not all triple GLP-1/GIP/GCG agonists are advancing to late-stage clinical trials. For instance, SAR441255—designed with balanced activity across all three receptors—showed some promising early efficacy for glycaemic control in a phase 1 single-dose study and was generally well tolerated [65], however, no further trials have been reported since 2019.

### Other Combinations of Triple Agonism in Development

GLP1/GIP/GCG is the only combination of hormone receptor agonism under investigation as a unimolecular triple agonist in phase 2 or 3 trials for obesity. However, DR-10,624, a GLP-1/GCG/fibroblast growth factor 21 (FGF-21) triple agonist is under investigation for individuals with severe hypertriglyceridemia (not limited to individuals with obesity; NCT06555640). Indeed, there may be synergy between FGF-21 and GCG receptor agonism, due to their similar metabolic actions, particularly related to brown adipose tissue differentiation and activation [32], with FGF21 having additional actions in the liver, reducing hepatic lipogenesis and enhancing hepatic insulin sensitivity [66, 67].

Although all unimolecular triple agonists in phase 2 trials for obesity are GLP1/GIP/GCG triple agonists, there is an ongoing phase 2 clinical trial of bioglutide, an oral insulin-like-growth factor-1 (IGF-1)/GLP-1/GIP/GCG quadruple agonist (NCT06564753) for individuals with obesity, which is also being trialled in combination with tirzepatide (NCT06643728). However, triple receptor agonism, or agonism/antagonism combinations, can be achieved not only though unimolecular triple agonists, but also by combining mono- and dual-agonists. According to ClinicalTrials.gov, several bimolecular combinations alongside the dual GLP-1/GIP agonist tirzepatide are currently under investigation in phase 2 trials including eloralintide (NCT06603571), an amylin receptor agonist; mibavademab, a leptin receptor agonist (NCT06373146); and bremelanotide, a melanocortin 4 receptor agonist (NCT06565611). Given that several further dual agonists are coming closer to becoming licenced, such as mazdutide and survodutide, with many more in development [15], combining licenced dual agonists with developing mono-agonists may be a faster way to develop effective treatments achieving triple agonism.

### Challenges in Developing Multi-Receptor Agonists

In the era of multi-receptor agonists and increasingly personalised medicine, striking the balance between the relative potency of agonists across various receptors poses a challenge. The ratio of target-receptor agonism can be manipulated to maximise efficacy whilst optimising adverse effects [31]. However, the effects of combinations of multi-receptor agonists in humans can be challenging to predict from animal studies [68]. Furthermore, it is likely that the optimal ratio of receptor agonism across target receptors varies between individuals. Combining mono- and/or dual-agonists to achieve triple agonism may allow for personalisation of treatment, aiming to find the optimal balance of receptor agonism for a specific individual. The challenge facing this strategy is that monotherapies often have varying pharmacokinetics and pharmacodynamics including half-life [69], meaning that the balance of target-receptor agonism may vary over time when using two different agents with varying profiles. Artificial Intelligence may allow for more accurate personalisation of therapy to predict the optimum ‘cocktail’ of mono agonist therapies based on the genetics and metabolomics of an individual and may quicken the process of drug development [70, 71].

## Conclusions

Despite recent advances in obesity pharmacotherapies, the need for improvements in medication efficacy, whilst minimising adverse effects, remains. Triple receptor agonists have the potential to further bridge the efficacy gap in WL between BS and the currently approved obesity pharmacotherapies, with the added potential for other synergistic metabolic benefits, such as improving glycaemia in people with T2D, and treating or preventing MASLD/MASH. Retatrutide is the unimolecular triple agonist furthest in development and data from phase 2 trials suggests that this may soon become the most potent weight-lowering pharmacotherapy to be licenced. Although data from early phase trials suggest that retatrutide is well tolerated with a similar adverse event profile to GLP-1 RAs, additional research assessing long-term safety and efficacy of retatrutide in people living with obesity and/or T2D (including cardiovascular outcomes) is awaited over the next five years.

## Key References

- Jastreboff AM, Kaplan LM, Frías JP, Wu Q, Du Y, Gurbuz S, et al. Triple–Hormone-Receptor Agonist Retatrutide for Obesity — A Phase 2 Trial. N Engl J Med. 2023 Aug 10;389(6):514–26.
-
  - This is the phase 2 trial of retatrutide in individuals with obesity (without type 2 diabetes) in which much of the discussion of this review is focused. Most notably, the highest dose of retatrutide (12 mg) led to 24.2% weight loss (vs 2.1% with placebo) at 48 weeks.
- Rosenstock J, Frias J, Jastreboff AM, Du Y, Lou J, Gurbuz S, et al. Retatrutide, a GIP, GLP-1 and glucagon receptor agonist, for people with type 2 diabetes: a randomised, double-blind, placebo and active-controlled, parallel-group, phase 2 trial conducted in the USA. The Lancet. 2023 Aug;402(10401):529–44.
-
  - This is the other phase 2 trial of retatrutide in individuals with type 2 diabetes and overweight/obesity. This trial the highest dose of retatrutide (12 mg) led to 16.9% weight loss (vs 3.0% with placebo) at 36 weeks.

## Data Availability

No datasets were generated or analysed during the current study.

## References

[1] Abdelaal M, le Roux CW, Docherty NG. Morbidity and mortality associated with obesity. Ann Transl Med. 2017;5(7):161. 28480197 10.21037/atm.2017.03.107 PMC5401682

[2] Ram Sohan P, Mahakalkar C, Kshirsagar S, Bikkumalla S, Reddy S, Hatewar A, et al. Long-Term effectiveness and outcomes of bariatric surgery: A comprehensive review of current evidence and emerging trends. Cureus. 2024;16(8):e66500. 39247032 10.7759/cureus.66500 PMC11381104

[3] Sjöström L, Narbro K, Sjöström CD, Karason K, Larsson B, Wedel H, et al. Effects of bariatric surgery on mortality in Swedish obese subjects. N Engl J Med. 2007;357(8):741–52. 17715408 10.1056/NEJMoa066254

[4] Drucker DJ. Mechanisms of action and therapeutic application of Glucagon-like Peptide-1. Cell Metab. 2018;27(4):740–56. 29617641 10.1016/j.cmet.2018.03.001

[5] Marso SP, Bain SC, Consoli A, Eliaschewitz FG, Jódar E, Leiter LA, et al. Semaglutide and cardiovascular outcomes in patients with type 2 diabetes. N Engl J Med. 2016;375(19):1834–44. 27633186 10.1056/NEJMoa1607141

[6] Marso SP, Daniels GH, Brown-Frandsen K, Kristensen P, Mann JFE, Nauck MA, et al. Liraglutide and cardiovascular outcomes in type 2 diabetes. N Engl J Med. 2016;375(4):311–22. 27295427 10.1056/NEJMoa1603827 PMC4985288

[7] Rossing P, Baeres FMM, Bakris G, Bosch-Traberg H, Gislum M, Gough SCL et al. The rationale, design and baseline data of FLOW, a kidney outcomes trial with once-weekly semaglutide in people with type 2 diabetes and chronic kidney disease. Nephrol Dial Transpl. 2023;gfad009. 10.1093/ndt/gfad009 PMC10469096 36651820

[8] Wilding JPH, Batterham RL, Calanna S, Davies M, Van Gaal LF, Lingvay I, et al. Once-Weekly semaglutide in adults with overweight or obesity. N Engl J Med. 2021;384(11):989–1002. 33567185 10.1056/NEJMoa2032183

[9] Akalestou E, Miras AD, Rutter GA, le Roux CW. Mechanisms of weight loss after obesity surgery. Endocr Rev. 2022;43(1):19–34. 34363458 10.1210/endrev/bnab022 PMC8755990

[10] Svane MS, Jørgensen NB, Bojsen-Møller KN, Dirksen C, Nielsen S, Kristiansen VB, et al. Peptide YY and glucagon-like peptide-1 contribute to decreased food intake after Roux-en-Y gastric bypass surgery. Int J Obes (Lond). 2016;40(11):1699–706. 27434221 10.1038/ijo.2016.121

[11] le Roux CW, Aylwin SJB, Batterham RL, Borg CM, Coyle F, Prasad V, et al. Gut hormone profiles following bariatric surgery favor an anorectic state, facilitate weight loss, and improve metabolic parameters. Ann Surg. 2006;243(1):108–14. 16371744 10.1097/01.sla.0000183349.16877.84 PMC1449984

[12] Nielsen MS, Ritz C, Wewer Albrechtsen NJ, Holst JJ, le Roux CW, Sjödin A. Oxyntomodulin and Glicentin May predict the effect of bariatric surgery on food preferences and weight loss. J Clin Endocrinol Metab. 2020;105(4):dgaa061. 32016415 10.1210/clinem/dgaa061

[13] Perakakis N, Kokkinos A, Peradze N, Tentolouris N, Ghaly W, Pilitsi E, et al. Circulating levels of Gastrointestinal hormones in response to the most common types of bariatric surgery and predictive value for weight loss over one year: evidence from two independent trials. Metabolism. 2019;101:153997. 31672446 10.1016/j.metabol.2019.153997

[14] Albaugh VL, Banan B, Ajouz H, Abumrad NN, Flynn CR. Bile acids and bariatric surgery. Mol Aspects Med. 2017;56:75–89. 28390813 10.1016/j.mam.2017.04.001 PMC5603298

[15] Melson E, Ashraf U, Papamargaritis D, Davies MJ. What is the pipeline for future medications for obesity? Int J Obes (Lond). 2024. 10.1038/s41366-024-01473-y PMC11971045 38302593

[16] Nørregaard PK, Deryabina MA, Tofteng Shelton P, Fog JU, Daugaard JR, Eriksson PO, et al. A novel GIP analogue, ZP4165, enhances glucagon-like peptide-1-induced body weight loss and improves glycaemic control in rodents. Diabetes Obes Metab. 2018;20(1):60–8. 28598027 10.1111/dom.13034

[17] Cegla J, Troke RC, Jones B, Tharakan G, Kenkre J, McCullough KA, et al. Coinfusion of low-dose GLP-1 and glucagon in man results in a reduction in food intake. Diabetes. 2014;63(11):3711–20. 24939425 10.2337/db14-0242

[18] Pocai A, Carrington PE, Adams JR, Wright M, Eiermann G, Zhu L, et al. Glucagon-Like peptide 1/glucagon receptor dual agonism reverses obesity in mice. Diabetes. 2009;58(10):2258–66. 19602537 10.2337/db09-0278 PMC2750209

[19] NamKoong C, Kim MS, Jang BT, Lee YH, Cho YM, Choi HJ. Central administration of GLP-1 and GIP decreases feeding in mice. Biochem Biophys Res Commun. 2017;490(2):247–52. 28610922 10.1016/j.bbrc.2017.06.031

[20] Finan B, Ma T, Ottaway N, Müller TD, Habegger KM, Heppner KM, et al. Unimolecular dual incretins maximize metabolic benefits in rodents, monkeys, and humans. Sci Transl Med. 2013;5(209):209ra151. 24174327 10.1126/scitranslmed.3007218

[21] Karagiannis T, Malandris K, Avgerinos I, Stamati A, Kakotrichi P, Liakos A, et al. Subcutaneously administered Tirzepatide vs semaglutide for adults with type 2 diabetes: a systematic review and network meta-analysis of randomised controlled trials. Diabetologia. 2024;67(7):1206–22. 38613667 10.1007/s00125-024-06144-1 PMC11153294

[22] Finan B, Yang B, Ottaway N, Smiley DL, Ma T, Clemmensen C, et al. A rationally designed monomeric peptide triagonist corrects obesity and diabetes in rodents. Nat Med. 2015;21(1):27–36. 25485909 10.1038/nm.3761

[23] Jall S, Sachs S, Clemmensen C, Finan B, Neff F, DiMarchi RD, et al. Monomeric GLP-1/GIP/glucagon triagonism corrects obesity, hepatosteatosis, and dyslipidemia in female mice. Mol Metab. 2017;6(5):440–6. 28462078 10.1016/j.molmet.2017.02.002 PMC5404097

[24] Nauck MA, Quast DR, Wefers J, Pfeiffer AFH. The evolving story of incretins (GIP and GLP-1) in metabolic and cardiovascular disease: A pathophysiological update. Diabetes Obes Metabolism. 2021;23(S3):5–29. 10.1111/dom.14496 34310013

[25] Borner T, Geisler CE, Fortin SM, Cosgrove R, Alsina-Fernandez J, Dogra M, et al. GIP receptor agonism attenuates GLP-1 receptor Agonist-Induced nausea and Emesis in preclinical models. Diabetes. 2021;70(11):2545–53. 34380697 10.2337/db21-0459 PMC8564411

[26] Knop FK, Urva S, Rettiganti M, Benson CT, Roell WC, Mather KJ, et al. A long-acting glucose-dependent insulinotropic polypeptide receptor agonist improves the Gastrointestinal tolerability of glucagon-like peptide-1 receptor agonist therapy. Diabetes Obes Metab. 2024;26(11):5474–8. 39188238 10.1111/dom.15875

[27] Jastreboff AM, Aronne LJ, Ahmad NN, Wharton S, Connery L, Alves B, et al. Tirzepatide once weekly for the treatment of obesity. N Engl J Med. 2022;387(3):205–16. 35658024 10.1056/NEJMoa2206038

[28] Malhotra A, Grunstein RR, Fietze I, Weaver TE, Redline S, Azarbarzin A, et al. Tirzepatide for the treatment of obstructive sleep apnea and obesity. N Engl J Med. 2024;391(13):1193–205. 38912654 10.1056/NEJMoa2404881 PMC11598664

[29] Loomba R, Hartman ML, Lawitz EJ, Vuppalanchi R, Boursier J, Bugianesi E et al. Tirzepatide for metabolic Dysfunction-Associated steatohepatitis with liver fibrosis. N Engl J Med. 2024. 10.1056/NEJMoa2401943 38856224

[30] Packer M, Zile MR, Kramer CM, Baum SJ, Litwin SE, Menon V, et al. Tirzepatide for heart failure with preserved ejection fraction and obesity. N Engl J Med. 2025;392(5):427–37. 39555826 10.1056/NEJMoa2410027

[31] Hope DCD, Vincent ML, Tan TMM. Striking the balance: GLP-1/Glucagon Co-Agonism as a treatment strategy for obesity. Front Endocrinol (Lausanne). 2021;12:735019. 34566894 10.3389/fendo.2021.735019 PMC8457634

[32] Hope DCD, Tan TMM. Glucagon and energy expenditure; revisiting amino acid metabolism and implications for weight loss therapy. Peptides. 2023;162:170962. 36736539 10.1016/j.peptides.2023.170962

[33] Salter JM, Davidson IW, Best CH. The pathologic effects of large amounts of glucagon. Diabetes. 1957;6(3):248–52. 13427630 10.2337/diab.6.3.248

[34] Tan TM, Field BCT, McCullough KA, Troke RC, Chambers ES, Salem V, et al. Coadministration of glucagon-like peptide-1 during glucagon infusion in humans results in increased energy expenditure and amelioration of hyperglycemia. Diabetes. 2013;62(4):1131–8. 23248172 10.2337/db12-0797 PMC3609580

[35] Biologics I. Innovent presents the results of the first phase 3 study of mazdutide for weight management at the ADA’s 84th scientific sessions [Internet]. [cited 2025 Mar 20]. Available from: https://www.prnewswire.com/news-releases/innovent-presents-the-results-of-the-first-phase-3-study-of-mazdutide-for-weight-management-at-the-adas-84th-scientific-sessions-302180995.html

[36] le Roux CW, Steen O, Lucas KJ, Startseva E, Unseld A, Hennige AM. Glucagon and GLP-1 receptor dual agonist survodutide for obesity: a randomised, double-blind, placebo-controlled, dose-finding phase 2 trial. Lancet Diabetes Endocrinol. 2024;12(3):162–73. 38330987 10.1016/S2213-8587(23)00356-X

[37] Newsome PN, Ambery P. Incretins (GLP-1 receptor agonists and dual/triple agonists) and the liver. J Hepatol. 2023;79(6):1557–65. 37562748 10.1016/j.jhep.2023.07.033

[38] Guzman CB, Zhang XM, Liu R, Regev A, Shankar S, Garhyan P, et al. Treatment with LY2409021, a glucagon receptor antagonist, increases liver fat in patients with type 2 diabetes. Diabetes Obes Metab. 2017;19(11):1521–8. 28371155 10.1111/dom.12958

[39] Winther JB, Holst JJ. Glucagon agonism in the treatment of metabolic diseases including type 2 diabetes mellitus and obesity. Diabetes Obes Metab. 2024;26(9):3501–12. 38853300 10.1111/dom.15693

[40] Romero-Gómez M, Lawitz E, Shankar RR, Chaudhri E, Liu J, Lam RLH, et al. A phase IIa active-comparator-controlled study to evaluate the efficacy and safety of Efinopegdutide in patients with non-alcoholic fatty liver disease. J Hepatol. 2023;79(4):888–97. 37355043 10.1016/j.jhep.2023.05.013

[41] Sanyal Arun J, Pierre B, Mandy F, Neff Guy W, Eric L, Elisabetta B et al. A phase 2 randomized trial of survodutide in MASH and fibrosis. N Engl J Med. 2024 Jul 25;391(4):311-319.10.1056/NEJMoa2401755 10.1056/NEJMoa2401755 38847460

[42] Coskun T, Urva S, Roell WC, Qu H, Loghin C, Moyers JS, et al. LY3437943, a novel triple glucagon, GIP, and GLP-1 receptor agonist for glycemic control and weight loss: from discovery to clinical proof of concept. Cell Metab. 2022;34(9):1234–e12479. 35985340 10.1016/j.cmet.2022.07.013

[43] Urva S, Coskun T, Loh MT, Du Y, Thomas MK, Gurbuz S, et al. LY3437943, a novel triple GIP, GLP-1, and glucagon receptor agonist in people with type 2 diabetes: a phase 1b, multicentre, double-blind, placebo-controlled, randomised, multiple-ascending dose trial. Lancet. 2022;400(10366):1869–81. 36354040 10.1016/S0140-6736(22)02033-5

[44] Urva S, O’Farrell L, Du Y, Loh MT, Hemmingway A, Qu H, et al. The novel GIP, GLP-1 and glucagon receptor agonist Retatrutide delays gastric emptying. Diabetes Obes Metab. 2023;25(9):2784–8. 37311727 10.1111/dom.15167

[45] Jastreboff AM, Kaplan LM, Frías JP, Wu Q, Du Y, Gurbuz S, et al. Triple–Hormone-Receptor agonist Retatrutide for Obesity — A phase 2 trial. N Engl J Med. 2023;389(6):514–26. 37366315 10.1056/NEJMoa2301972

[46] Rosenstock J, Frias J, Jastreboff AM, Du Y, Lou J, Gurbuz S, et al. Retatrutide, a GIP, GLP-1 and glucagon receptor agonist, for people with type 2 diabetes: a randomised, double-blind, placebo and active-controlled, parallel-group, phase 2 trial conducted in the USA. Lancet. 2023;402(10401):529–44. 37385280 10.1016/S0140-6736(23)01053-X

[47] Sanyal AJ, Kaplan LM, Frias JP, Brouwers B, Wu Q, Thomas MK, et al. Triple hormone receptor agonist Retatrutide for metabolic dysfunction-associated steatotic liver disease: a randomized phase 2a trial. Nat Med. 2024;30(7):2037–48. 38858523 10.1038/s41591-024-03018-2 PMC11271400

[48] Heerspink HJL, Lu Z, Du Y, Duffin KL, Coskun T, Haupt A et al. The effect of retatrutide on kidney parameters in participants with type 2 diabetes and/or obesity. Kidney Int Rep. 2025 Apr 2;10(6):1980-1992. 10.1016/j.ekir.2025.03.049 10.1016/j.ekir.2025.03.049 PMC12231004 40630318

[49] Davies M, Færch L, Jeppesen OK, Pakseresht A, Pedersen SD, Perreault L, et al. Semaglutide 2·4 mg once a week in adults with overweight or obesity, and type 2 diabetes (STEP 2): a randomised, double-blind, double-dummy, placebo-controlled, phase 3 trial. Lancet. 2021;397(10278):971–84. 33667417 10.1016/S0140-6736(21)00213-0

[50] Garvey WT, Frias JP, Jastreboff AM, le Roux CW, Sattar N, Aizenberg D, et al. Tirzepatide once weekly for the treatment of obesity in people with type 2 diabetes (SURMOUNT-2): a double-blind, randomised, multicentre, placebo-controlled, phase 3 trial. Lancet. 2023;402(10402):613–26. 37385275 10.1016/S0140-6736(23)01200-X

[51] Novo Nordisk [Internet]. [cited 2025 Mar 6]. News Details. Available from: https://www.novonordisk.com/content/nncorp/global/en/news-and-media/news-and-ir-materials/news-details.html

[52] Zhang B, Cheng Z, Chen J, Zhang X, Liu D, Jiang H, et al. Efficacy and safety of mazdutide in Chinese patients with type 2 diabetes: A randomized, Double-Blind, Placebo-Controlled phase 2 trial. Diabetes Care. 2024;47(1):160–8. 37943529 10.2337/dc23-1287 PMC10733643

[53] Boehringer Ingelheim A, Phase II, Randomized P, Group. Dose-finding study of subcutaneously administered BI 456906 for 16 weeks, compared with placebo and open-label semaglutide in patients with type 2 diabetes mellitus. [Internet]. clinicaltrials.gov; 2022 Nov [cited 2025 Mar 21]. Report No.: NCT04153929. Available from: https://clinicaltrials.gov/study/NCT04153929

[54] Sjöström L, Peltonen M, Jacobson P, Ahlin S, Andersson-Assarsson J, Anveden Å, et al. Association of bariatric surgery with long-term remission of type 2 diabetes and with microvascular and macrovascular complications. JAMA. 2014;311(22):2297–304. 24915261 10.1001/jama.2014.5988

[55] Goldney J, Davies MJ. GLP1 agonists: current and future landscape of clinical trials for patients with metabolic dysfunction. Nat Rev Gastroenterol Hepatol. 2024. 10.1038/s41575-024-00977-2 39242961

[56] Habegger KM, Heppner KM, Geary N, Bartness TJ, DiMarchi R, Tschöp MH. The metabolic actions of glucagon revisited. Nat Rev Endocrinol. 2010;6(12):689–97. 20957001 10.1038/nrendo.2010.187 PMC3563428

[57] Perkovic V, Tuttle KR, Rossing P, Mahaffey KW, Mann JFE, Bakris G, et al. Effects of semaglutide on chronic kidney disease in patients with type 2 diabetes. N Engl J Med. 2024;391(2):109–21. 38785209 10.1056/NEJMoa2403347

[58] Lincoff AM, Brown-Frandsen K, Colhoun HM, Deanfield J, Emerson SS, Esbjerg S, et al. Semaglutide and cardiovascular outcomes in obesity without diabetes. N Engl J Med. 2023;389(24):2221–32. 37952131 10.1056/NEJMoa2307563

[59] Boden G, Rezvani I, Owen OE. Effects of glucagon on plasma amino acids. J Clin Invest. 1984;73(3):785–93. 6142902 10.1172/JCI111272 PMC425081

[60] Nair KS, Halliday D, Matthews DE, Welle SL. Hyperglucagonemia during insulin deficiency accelerates protein catabolism. Am J Physiology-Endocrinology Metabolism. 1987;253(2):E208–13. 10.1152/ajpendo.1987.253.2.E208 3303968

[61] Almdal TP, Heindorff H, Bardram L, Vilstrup H. Increased amino acid clearance and Urea synthesis in a patient with Glucagonoma. Gut. 1990;31(8):946–8. 2167278 10.1136/gut.31.8.946 PMC1378630

[62] Mallinson CN, Bloom SR, Warin AP, Salmon PR, Cox B. A Glucagonoma syndrome. Lancet. 1974;2(7871):1–5. 4134714 10.1016/s0140-6736(74)91343-9

[63] Hope DCD, Hinds CE, Lopes T, Vincent ML, Shrewsbury JV, Yu ATC, et al. Hypoaminoacidemia underpins glucagon-mediated energy expenditure and weight loss. Cell Rep Med. 2022;3(11):100810. 36384093 10.1016/j.xcrm.2022.100810 PMC9729826

[64] Altimmune Presents Data from Phase 2 MOMENTUM Trial of Pemvidutide in Obesity during Oral Presentation at the American Diabetes Association. ’s 84th Scientific Sessions– Altimmune [Internet]. [cited 2025 Apr 7]. Available from: https://ir.altimmune.com/news-releases/news-release-details/altimmune-presents-data-phase-2-momentum-trial-pemvidutide

[65] Bossart M, Wagner M, Elvert R, Evers A, Hübschle T, Kloeckener T, et al. Effects on weight loss and glycemic control with SAR441255, a potent unimolecular peptide GLP-1/GIP/GCG receptor triagonist. Cell Metab. 2022;34(1):59–e7410. 34932984 10.1016/j.cmet.2021.12.005

[66] Falamarzi K, Malekpour M, Tafti MF, Azarpira N, Behboodi M, Zarei M. The role of FGF21 and its analogs on liver associated diseases. Front Med (Lausanne). 2022;9:967375. 36457562 10.3389/fmed.2022.967375 PMC9705724

[67] Jeong C, Han N, Jeon N, Rhee S, jin, Staatz CE, Kim MS, et al. Efficacy and safety of fibroblast growth Factor-21 analogs for the treatment of metabolic Dysfunction-Associated steatohepatitis: A systematic review and Meta-Analysis. Clin Pharmacol Ther. 2024;116(1):72–81. 38666606 10.1002/cpt.3278

[68] Dissanayake HA, Somasundaram NP. Polyagonists in type 2 diabetes management. Curr Diab Rep. 2024;24(1):1–12. 38150106 10.1007/s11892-023-01530-2

[69] Hasib A. Multiagonist unimolecular peptides for obesity and type 2 diabetes: current advances and future directions. Clin Med Insights Endocrinol Diabetes. 2020;13:1179551420905844. 32110131 10.1177/1179551420905844 PMC7025423

[70] BioSpace [Internet]. 2023 [cited 2025 Mar 6]. Mindrank announces first-in-human phase 1 study of MDR-001 in China. Available from: https://www.biospace.com/mindrank-announces-first-in-human-phase-1-study-of-mdr-001-in-china

[71] Gupta R, Srivastava D, Sahu M, Tiwari S, Ambasta RK, Kumar P. Artificial intelligence to deep learning: machine intelligence approach for drug discovery. Mol Divers. 2021;25(3):1315–60. 33844136 10.1007/s11030-021-10217-3 PMC8040371

